# Development and Application of a Lignin-Based Polyol for Sustainable Reactive Polyurethane Adhesives Synthesis

**DOI:** 10.3390/polym16131928

**Published:** 2024-07-06

**Authors:** Víctor M. Serrano-Martínez, Carlota Hernández-Fernández, Henoc Pérez-Aguilar, María Pilar Carbonell-Blasco, Avelina García-García, Elena Orgilés-Calpena

**Affiliations:** 1 Footwear Technology Centre, Campo Alto Campground, 03600 Alicante, Spainpcarbonell@inescop.es (M.P.C.-B.); eorgiles@inescop.es (E.O.-C.); 2 MCMA Group, Department of Inorganic Chemistry and Institute of Materials, University of Alicante, Ap. 99, 03080 Alicante, Spain; a.garcia@ua.es

**Keywords:** polyurethane adhesives, lignin-based polyols, sustainable synthesis, rice straw valorisation, hot melt adhesives (HMPUR), biopolymers, renewable resources

## Abstract

In response to the environmental impacts of conventional polyurethane adhesives derived from fossil fuels, this study introduces a sustainable alternative utilizing lignin-based polyols extracted from rice straw through a process developed at INESCOP. This research explores the partial substitution of traditional polyols with lignin-based equivalents in the synthesis of reactive hot melt polyurethane adhesives (HMPUR) for the footwear industry. The performance of these eco-friendly adhesives was rigorously assessed through Thermogravimetric Analysis (TGA), Differential Scanning Calorimetry (DSC), rheological analysis, and T-peel tests to ensure their compliance with relevant industry standards. Preliminary results demonstrate that lignin-based polyols can effectively replace a significant portion of fossil-derived polyols, maintaining essential adhesive properties and marking a significant step towards more sustainable adhesive solutions. This study not only highlights the potential of lignin in the realm of sustainable adhesive production but also emphasises the valorisation of agricultural by-products, thus aligning with the principles of green chemistry and sustainability objectives in the polymer industry.

## 1. Introduction

Traditional polyurethane adhesives are prevalent in various industries, including footwear, and are predominantly synthesised from polyols and isocyanates derived from fossil sources. These adhesives are non-renewable, contributing to a significant environmental impact due to their carbon footprint and sustainability issues [1,2,3]. The reliance on petrochemical sources to produce polyurethane highlights the urgent need for sustainable alternatives in adhesive synthesis, driven by global initiatives and regulations aimed at reducing environmental harm and promoting sustainability [4,5,6,7]. The environmental impacts of traditional polyurethane adhesives stem from their petrochemical origin, which is associated with greenhouse gas emissions and depletion of non-renewable resources. This has led to a growing interest in developing greener adhesives that minimise the use or generation of hazardous substances, decrease environmental impacts, and reduce energy consumption throughout their life cycle [8]. Efforts to develop more sustainable adhesives have included the exploration of bio-based polyols, such as those derived from lignin, vegetable oils, and other renewable resources; however, these efforts face challenges, including the need to match or exceed the performance of traditional adhesives and to ensure the economic viability of the greener alternatives [9,10,11]. Lignin-based polyols represent a promising avenue for adhesive innovation, due to lignin’s abundance as a by-product of the pulp and paper industry and its unique properties. Lignin is a natural aromatic polymer rich in functional hydroxyl groups, making it an attractive bio-based alternative to fossil-based polyols for the synthesis of polyurethanes. Despite its potential, lignin remains under-utilised in industrial applications, partly due to challenges in processing and modifying lignin to achieve the desired reactivity and performance in adhesive formulations [12,13,14]. Previous works have demonstrated the viability of lignin-based polyols in producing adhesives with comparable or even superior properties to their petroleum-based counterparts, addressing both performance and environmental sustainability concerns [5]. The exploration of lignin in adhesive innovation is not new, but its application has gained momentum due to the pressing need for sustainable material sources. The development of lignin-based polyols for PU adhesives represents a significant step towards reducing the environmental impact of adhesives, thus aligning with global sustainability goals and addressing the urgent need for renewable and less harmful materials in the adhesive industry [11].

Expanding on these developments, reactive polyurethane hot melt adhesives (HMPUR) stand out as a versatile and robust option that is gaining traction across various industrial and commercial sectors. At room temperature, these adhesives maintain a solid state and undergo curing upon exposure to moisture, which is facilitated by the reaction between the polyols and an excess of diisocyanates. This characteristic not only accelerates the setting process but also enhances the bond strength, making HMPUR ideal for applications demanding quick and durable adhesion. Adopting bio-based polyols, such as those derived from lignin, further reduces the environmental burden of these adhesives, supporting the transition towards more sustainable manufacturing practices. In addition to utilizing bio-based resources, recent research has also emphasised the integration of recycled polyols into HMPUR formulations. Existing studies have explored the feasibility of using bio-based polyols from vegetable sources, confirming their capability to meet the stringent quality standards for footwear applications while integrating a higher percentage of sustainable materials into adhesive formulations [14]. Moreover, a broader review of sustainable practices highlighted that materials such as lignin provide a significant hydroxyl functionality, which is crucial for polyurethane production, fostering the move towards a more circular economy through reducing reliance on petrochemicals [15]. Such advancements underscore the potential for integrating innovative and environmentally friendly materials into the industries traditionally dominated by petrochemical products.

Building upon this background, our previous study specifically introduced a novel process developed at INESCOP, employing a steam explosion method to extract lignin from rice straw waste—a significantly abundant agricultural by-product. This process is not only innovative, but also critical in addressing the urgent need for more sustainable waste management practices. The extracted lignin will be then transformed into polyols for the synthesis of HMPUR, particularly for use in the footwear industry. Our previous work demonstrated the feasibility of this method, which significantly enhances the accessibility and reactivity of lignin, facilitating its incorporation into polyol formulations with improved performance characteristics [16].

The objectives of this study are multifaceted. We aim to demonstrate that lignin-based polyols can effectively replace a substantial portion of fossil-based polyols without compromising the essential properties required for high-quality adhesives. This research also aligns with broader sustainability and green chemistry goals through valorising agricultural waste and reducing reliance on non-renewable resources [17,18,19,20]. Furthermore, the potential applications of these bio-based polyols in creating adhesives that meet industry standards for performance and environmental impact are explored.

In conclusion, the application of lignin-based polyols in the synthesis of reactive polyurethane adhesives presents a tangible step towards more sustainable material production. Through rigorous assessments, including Thermogravimetric Analysis (TGA), Differential Scanning Calorimetry (DSC), rheological analysis, and T-peel tests, this study aims to establish a new benchmark for the adhesive industry, contributing to its evolution towards sustainability.

## 2. Materials and Methods

### 2.1. Materials

For the synthesis of the lignin-based polyol, pure absolute ethanol (Panreac Appliquem, Barcelona, Spain; purity ≥ 99.8%, CAS 64-17-5), ethyl acetate (Quimidroga SA, Barcelona, Spain; CAS 141-78-6), and hydrochloric acid solution (ITW Reagents, Barcelona, Spain; 35%, CAS 7647-01-0) were used.

Regarding the synthesis of adhesives, conventional polyols as well as the more sustainable polyol obtained from lignin were used. This lignin-based polyol is denoted as LIGNOC (Mw = 929 g/mol, IOH = 182.25 mg kOH/g), which was developed at INESCOP; its properties were measured at the University of the Basque Country UPV/EHU. The 1,4-butanediol polyadipate (Hoopol F-580 Synthesia Technology, Barcelona, Spain; Mw = 3000 g/mol, IOH = 37–40 mgKOH/g) and polypropylene glycol (Quimidroga SA, Barcelona, Spain; Mw = 425 g/mol, IOH = 250–270 mgKOH/g) were used as polyols from fossil resources, and 4,4-diphenylmethane diisocyanate (MDI) (98% purity, Sigma Aldrich, Barcelona, Spain) was used as isocyanate.

### 2.2. Lignin-Based Polyol Synthesis Method from Rice Straw

Building on our prior work, this study utilised lignin derived from the supernatant of a steam explosion process designed for cellulose extraction from rice straw, as comprehensively detailed in [16]. This foundational process involves treating rice straw at 200 °C to facilitate the separation of cellulose and lignin, capturing the lignin in the supernatant.

**Figure 1:**
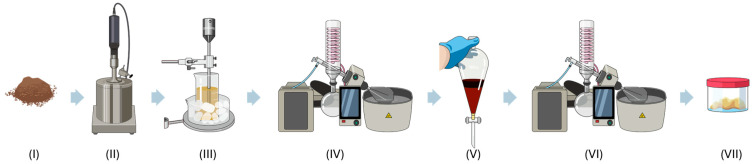
Lignin-based polyol synthesis process.

For the current study, 20 g of this lignin (step I) was dissolved in a 50/50 mixture of ethanol and water (60 mL of each) to promote solubilisation. The lignin raw material and subsequent processing steps are illustrated in Figure 1. After mixing, the lignin solution was subjected to organosolvent fractionation (step II) at 200 °C for 75 min to further break down its complex structure, called fractionation. The treated mixture was then vacuum filtered to separate the liquid fraction (black liquor) from the solids. The filtrate was processed through ultrasonication for one hour (step III), utilizing 35% of the power and 10 s on/off cycles, which helped to further degrade the lignin into smaller components. This step was crucial for preparing the lignin for subsequent chemical manipulation [21,22,23].

The black liquor was then subjected to rotary evaporation (step IV) in order to remove the ethanol, thus concentrating the lignin derivatives. The pH of this concentrated solution was adjusted to 2.5 using 6M hydrochloric acid. To enhance the separation of lignin derivatives, 80 mL of ethyl acetate was added. Phase separation was conducted using a separatory funnel (step V), where the ethyl acetate layer containing the lignin-derived polyol was carefully isolated.

The isolated ethyl acetate phase underwent a second rotary evaporation (step VI) to remove any residual solvent. The resultant polyol (step VII) was then purified by agitation in a washing bath to eliminate traces of ethyl acetate, thus ensuring its purity [24,25].

This refined method transforms lignin from rice straw into a high-value polyol suitable for producing HMPUR. The approach not only leverages rice straw, an agricultural by-product, as a feedstock for advanced material applications, but also exemplifies the scalability of our initial extraction process.

### 2.3. Reactive Polyurethane Hot Melt Adhesives (HMPURs) Synthesis Process

The synthesis process for reactive hot melt polyurethane adhesives can be affected by several factors, such as the molecular weight of the polyols used, the functionality of both polyols and isocyanate, or the stoichiometric ratio between the raw materials (NCO/OH).

Reactive polyurethane hot melt adhesives were synthesised using the prepolymer method [1,26], with an optimal NCO/OH index of 1.5.

**Figure 2:**
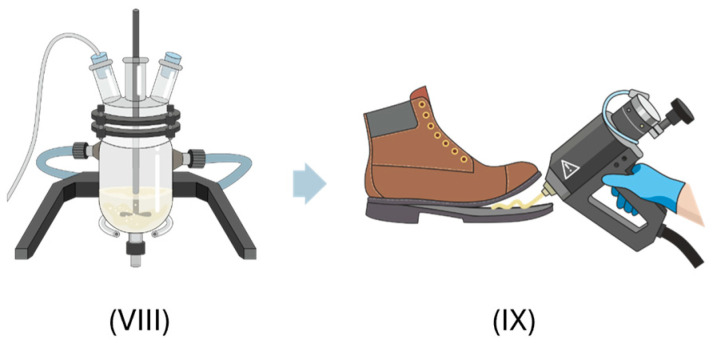
HMPURs synthesis process (VIII) and application of the adhesive (IX).

The procedure followed for the synthesis and application of the adhesives is shown in Figure 2. First, the polyols were melted and mixed at 90 °C in a glass reactor jacketed with thermal oil (step VIII), subjected to constant mechanical stirring of 300 rpm (Heidolph RZR 2021, Kel-heim, Germany) in a nitrogen atmosphere, according to previous research [14]. The isocyanate was then added. The progress of the reaction was monitored by determining the percentage of free NCO through the dibutylamine titration method [27]. Once the desired percentage of free isocyanate had been reached (Table 1), the reaction was stopped and the HMPUR adhesive was transferred in a melted state to a hermetically sealed cartridge, where the reaction continued for 18 h (annealing process). The airtight cartridge allowed for the subsequent application of the adhesive by means of a manual heat gun (step IX).

In the synthesis of HMPUR, the reference formula employed an equal mixture of 1,4-butanediol polyadipate and polypropylene glycol (PPG). A key component of this study involves conducting multiple syntheses in which this PPG is partially substituted with the polyol synthesised from rice straw in proportions of 2.5%, 5%, and 7.5%, as detailed in Table 1.

### 2.4. Characterisation Techniques

#### 2.4.1. Fourier Transform Infrared Spectroscopy (FTIR)

The chemical structure was studied using a Varian 660-IR (Varian Australia PTY LTD, Mulgrave, Australia) with a diamond prism. A total of 16 scans were averaged, with a resolution of 4 cm^−1^, the range analysed being from 500 to 4000 cm^−1^ with attenuated total reflection (ATR) technology [28].

#### 2.4.2. Thermogravimetric Analysis (TGA)

The thermal stability was evaluated using a TGA 2 STARe System thermal balance, equipped with the STARe v16.4 software from Mettler-Toledo, Switzerland. A sample size of approximately 7 to 10 mg of the adhesive was placed into an alumina crucible. The sample was then heated from 30 to 600 °C at a rate of 10 °C/min under an inert nitrogen atmosphere, with a nitrogen flow rate of 30 mL/min [29,30].

#### 2.4.3. Differential Scanning Calorimetry (DSC)

The thermal behaviour was studied using a DSC3 + STARe Systems calorimeter from Mettler-Toledo AG, Schwerzenbach, Switzerland. The experiments used samples weighing between 9 and 12 mg placed in aluminium pans, and were carried out in an inert nitrogen atmosphere with a flow rate of 30 mL/min and a temperature change rate of 10 °C/min. The analysis included two sequential runs: (i) Initial heating from −15 °C to 110 °C, followed by isothermal maintenance at 110 °C for three minutes to remove the sample’s thermal history, and (ii) a second heating phase from −65 °C to 100 °C, succeeded by isothermal cooling at 25 °C for 45 min. The optimal conditions for these DSC experiments were previously determined by the authors in earlier research [31].

#### 2.4.4. Rheological Analysis

The viscoelastic properties were determined on a Kinexus Pro+ rheometer (Malvern Panalytical, Malvern, UK). A 25 mm diameter aluminium top plate was used, on which 50 mL of the sample was placed.

For the polyol, a gap of 0.1 mm was set between the plates, and the tests were carried out with a shear rate sweep from 0.01 to 1000 s^−1^ (50 °C). For the adhesives, a gap of 0.4 mm was set, and a cooling sweep was performed from 160 to 25 °C, maintaining a cooling rate of 5 °C/min and using a constant frequency of 1 Hz at a deformation equal to 1%. 

#### 2.4.5. T-Peel Strength Test

The adhesion properties were assessed using the method outlined in the EN 1392:2007 standard [32]. For this evaluation, in addition to the reference adhesive and the newly synthesised adhesives, reference materials commonly used in footwear were used as substrates for the adhesive bonds. A medium-hard vulcanised styrene–butadiene rubber SBR-2 was used as the reference sole material, while chrome-tanned split leather was used as the upper reference material. Both materials were supplied by Proyección Europlan XXI S.L. and were die-cut into 150 × 30 mm specimens.

Prior to forming the joints, each material underwent specific surface treatments. The split leather samples were roughened using a P100 aluminium oxide abrasive cloth (Due Emme Abrasivi, Pavia, Italy) at 2800 rpm on a roughing machine from Superlema S.A. (Zaragoza, Spain). The SBR-2 rubber was roughened and then halogenated using a 2 wt% trichloroisocyanuric acid solution in ethyl acetate. The upper to sole adhesive joints, measuring 150 × 30 mm, were then prepared by bonding leather/HMPUR/SBR rubber.

The joint formation process began 30 min after applying the adhesive, where both adhesive films were activated by exposing them to infrared radiation at 80 °C in a CAN 02/01 temperature-controlled heater from AC&N (Elda, Spain). Immediately after, the materials were pressed together under a pressure of 1.8 bar for 10 s to ensure a strong bond. The adhesive joints were then conditioned at 23 °C and 50% relative humidity for 72 h. T-peel strength was finally evaluated using an Instron 34TM-10 universal testing machine (Instron Ltd., Buckinghamshire, UK) at a cross-head speed of 100 mm/min [33]. The bond strength values were calculated based on the width and scale of the test pieces, leading to an average and typical deviation calculated from five samples per joint. Failure modes were assessed according to ISO standard 17708 [34].

## 3. Results and Discussion

Throughout this section, the experimental results obtained are precisely described, and their interpretation and discussion are presented. With reference to the characterisation, the lignin polyol obtained was analysed through DSC, TGA, and FTIR, while the adhesives were characterised using FTIR, DSC, TGA, rheology, and T-peel strength tests.

### 3.1. Polyol Characterisation

**Figure 3:**
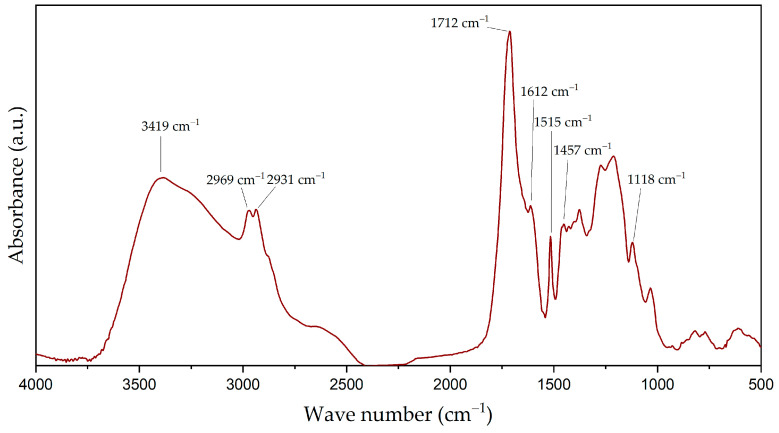
Infrared spectra of the polyol based on lignin obtained from rice straw.

After the polyol synthesis, an experimental characterisation of the product was carried out. The chemical composition was first studied using FTIR, with the spectra obtained shown in Figure 3.

The FTIR-ATR spectra illustrated in Figure 3 reveal distinct absorption bands that are indicative of various functional groups inherent to the lignin structure and the modifications involved in polyol synthesis. The spectrum displayed a broad absorption band in the region of 3500–3300 cm^−1^, related to the stretching vibration O-H of the hydroxyl group, common in polyols. Furthermore, 2969 and 2931 cm^−1^ peaks were assigned to the vibrations of aliphatic C-H bonds of methylene and methyl groups, indicating the presence of saturated hydrocarbon chains [35].

The absorption peak at 1712 cm^−1^ was identified as corresponding to carbonyl (C=O) stretching in unconjugated ketonic or ester groups, which are often introduced in lignin during the oxidative modification processes [36].

Finally, the phenylpropane skeleton of lignin—a fundamental component of its macromolecular structure—was evidenced by sharp and distinct bands at 1612, 1515, and 1457 cm^−1^. These bands are typically assigned to the vibrations of C=C bonds within the aromatic rings, confirming the retention of aromatic structures within the polyol. Furthermore, the stretching vibrations of C-O bonds with a specific peak at 1118 cm^−1^ is associated with C-O in secondary alcohols and aliphatic ethers [23]. The FTIR-ATR spectral data corroborate the chemical structure of the lignin-derived polyol, providing insight into the functional groups that contribute to its application potential in various polymeric and material science contexts.

**Figure 4:**
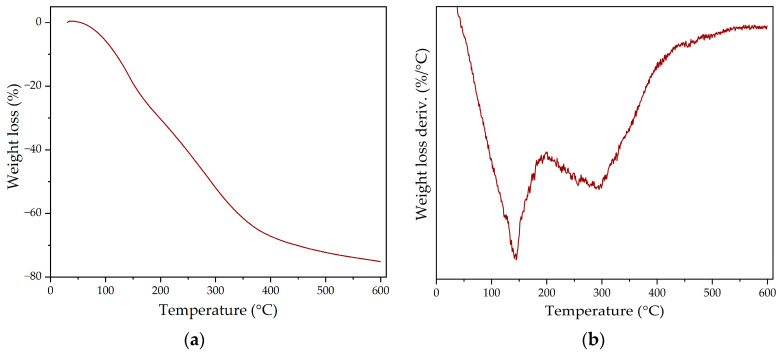
TGA results for the developed lignin-based polyol: (**a**) weight loss; (**b**) weight loss derivate.

The thermal stability of the polyol was then evaluated through TGA. Figure 4a shows the weight loss obtained, and its derivate is shown in Figure 4b; the results are collected in Table 2.

Regarding the weight loss derivate, it is possible to understand how the thermal degradation takes place in two different areas. The first area—between 100–200 °C—shows a maximum degradation peak at 140 °C, which could be assigned to the most volatile compounds present in the polyol. Meanwhile, the second degradation area appears between 200–500 °C, with a maximum peak at 302 °C associated with the degradation of lignin, as well as other oligomers present in the structure of the polyol [21].

**Figure 5:**
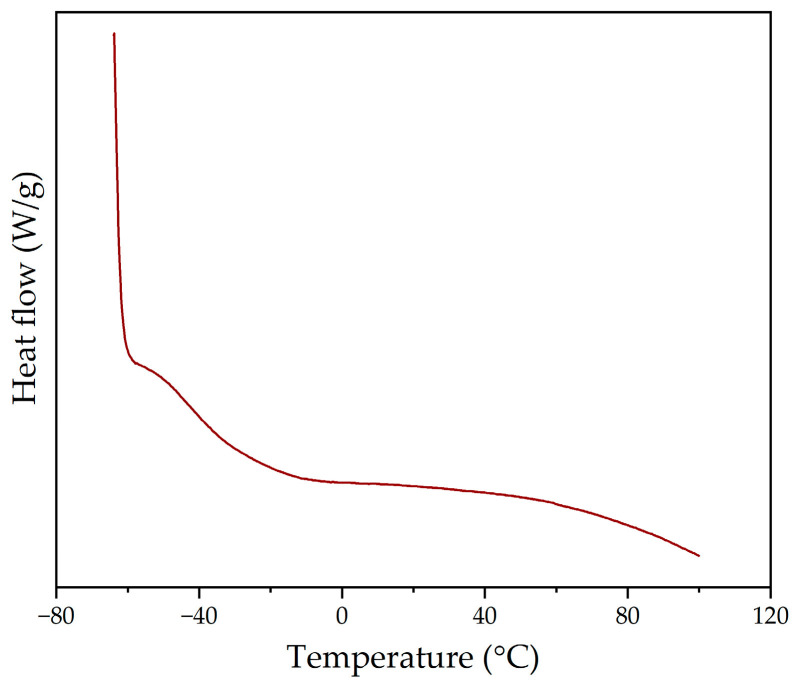
DSC curve of the lignin-based polyol during the second heating sweep.

In this way, the thermal properties of the polyol were also evaluated by DSC. The DSC curve obtained in the second heating sweep is shown in Figure 5, from which it can be observed that the polyol presents an amorphous state, with a single glass transition temperature of −40 °C [37].

**Figure 6:**
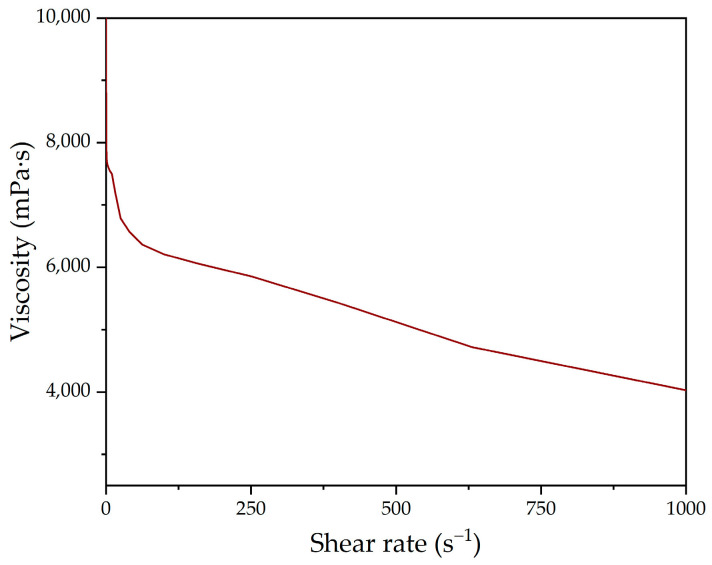
Viscosity of the lignin-based polyol obtained by plate–plate rheometric analysis.

Finally, plate–plate rheometry was used to determine the viscosity of the polyol—a parameter that represents its flow resistance. Figure 6 shows the result obtained in the shear rate sweep, from which it can be observed that the polyol presents a non-Newtonian pseudoplastic behaviour, decreasing its viscosity as the shear stress increases [38,39,40].

### 3.2. HMPURs Synthesis and Characterisation

The reaction time and free isocyanate (%NCO) measurement are crucial aspects in the synthesis of polyurethane adhesives, as detailed in Table 3, which presents the reaction conditions. Accurately measuring the reaction time is vital as it directly influences the final properties of the adhesive, such as viscosity, strength, and flexibility. In addition, the amount of free NCO at the end of the process is a key indicator of the reactivity and complete conversion of the precursors into the final product. Monitoring these parameters allows for the optimisation of the synthesis process to obtain polyurethane adhesives with desirable characteristics, ensuring consistent, high-quality performance in the final applications.

The values shown in Table 3 reflect the high reactivity of the developed lignin polyol, obtaining a reduction in reaction time of more than 50% for the synthesis with the lowest substitution and reaching a reduction of 85% in the synthesis time for the highest PPG substitution. Otherwise, the final free isocyanate obtained was almost constant in all of the adhesives.

**Figure 7:**
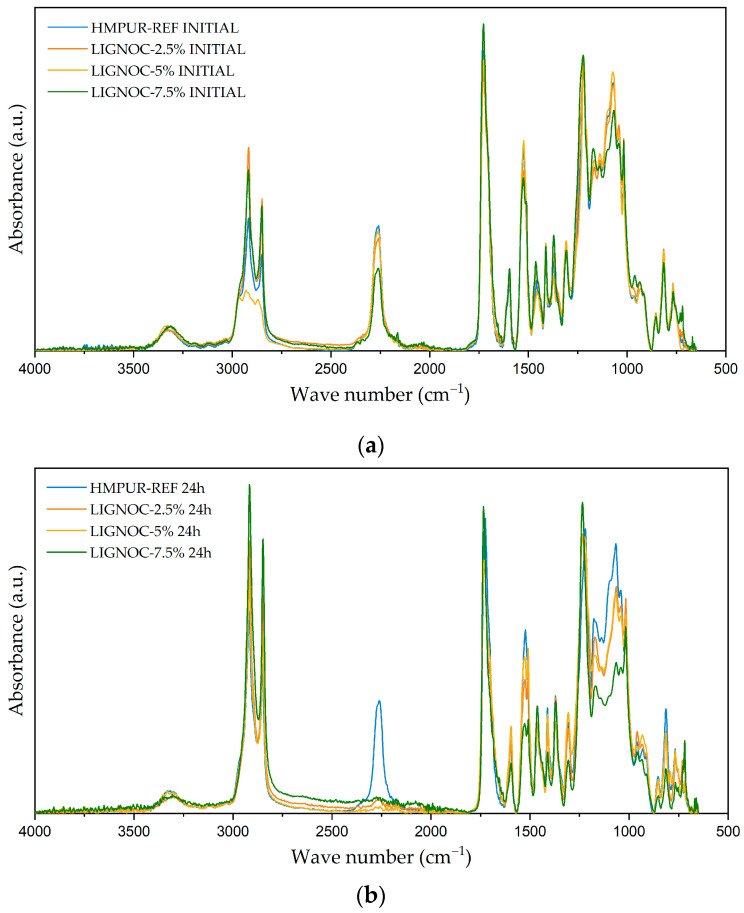
Comparison of the infrared spectra of applied adhesives: (**a**) infrared spectrum obtained immediately after application; and (**b**) infrared spectrum obtained after 24 h from application.

Regarding the adhesive characterisation, first, the chemical composition of the adhesives was studied using FTIR. Figure 7a shows the spectra obtained for the recently applied adhesive, from which it can be seen that the band corresponding to free -NCO appears in all cases for a wavelength of ~2250 cm^−1^. This band is characteristic of uncured polyurethane adhesives, and it was observed that the incorporation of the lignin-based polyol did not produce the appearance of any new bands in the spectra analysed, when compared with the reference HMPUR. Figure 7b shows the spectra obtained after 24 h of adhesive application, with a decrease in the band corresponding to the free -NCO in the case of adhesives with lignin-based polyols, corresponding to the consumption of free -NCO during the curing reaction with ambient moisture and, thus, showing much faster curing compared with the reference adhesive.

**Figure 8:**
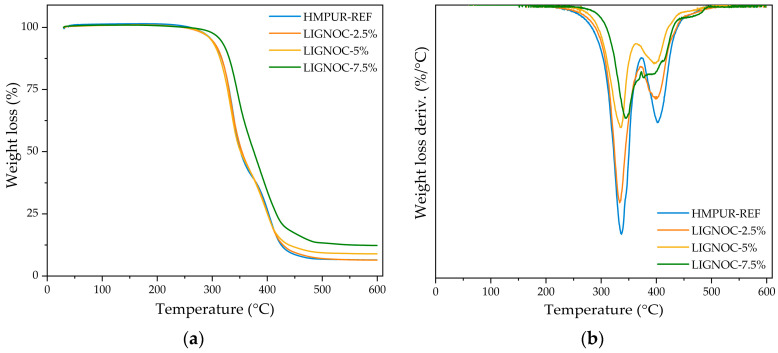
Comparison of the TGA results for the adhesives: (**a**) comparison of the samples’ weight loss; and (**b**) comparison of the samples’ weight loss derivate.

The thermal stability of the adhesives was then evaluated through TGA. Figure 8a,b show the comparison of the mass loss obtained for each sample and its derivate, respectively. All of the numerical results are collected in Table 4.

The data collected in Table 4 suggest that the addition of lignin-based polyol to the adhesives did not affect the thermal stability of the formulations with lower concentrations (LIGNOC-2.5% and LIGNOC-5%), maintaining thermal decomposition parameters comparable to the reference adhesive. However, the formulation with the highest substitution (LIGNOC-7.5%) demonstrated a marked improvement in thermal stability, evidenced by an increase in the first decomposition temperature, as well as a higher residue content. This increase in thermal stability and post-decomposition residue percentage can be attributed to the aromatic structure of lignin and its inherent thermal resistance, which provides greater structural integrity to the adhesive under high-temperature conditions [39,41,42]. Furthermore, observing Figure 8b, it is possible to verify that the ratio between hard segments—coming from isocyanate and low molecular weight polyols (decomposition above 338–400 °C)—and soft segments—coming from high molecular weight polyols (decomposition between 400–416 °C)—was maintained.

Thus, a slower decomposition rate is observed as the concentration of incorporated lignin-based polyol increases, suggesting that a higher content of it in the soft segments promotes a more stable matrix at high temperatures, which is critical for applications demanding high heat resistance in polyurethane adhesives [43,44].

**Figure 9:**
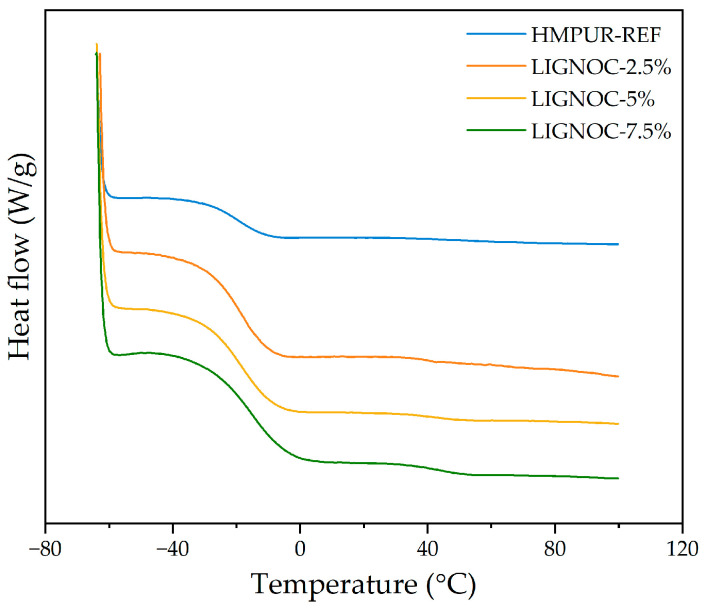
Comparison of the DSC curves of the HMPUR adhesives during the second heating sweep.

Thereafter, the thermal behaviour of the adhesives was evaluated using DSC. Figure 9 shows the DSC curve corresponding to the second heating sweep, obtained after eliminating the thermal history of the adhesives during the first temperature sweep. The obtained glass transition temperature (Tg) values are included in Table 5.

The reference adhesive shows a glass transition temperature around −19 °C, while the substitution of polypropylene glycol with the lignin-based polyol resulted in an increase in Tg for all cases. These results imply that even a small amount of lignin-based polyol acts to stiffen the adhesive matrix. This effect becomes more pronounced with higher concentrations of lignin-based polyol, observing that it contributes significantly to the rigidity of the adhesive, increasing the glass transition temperature even more at the highest substitution. Overall, these observations indicate that the polyol used predominantly influences the amorphous regions of the polymer, leading to progressively higher glass transition temperatures as the concentration of lignin increases, potentially due to increased interactions between polymer chains or alterations in the polymer structure introduced by the lignin-based polyol [7,45].

**Figure 10:**
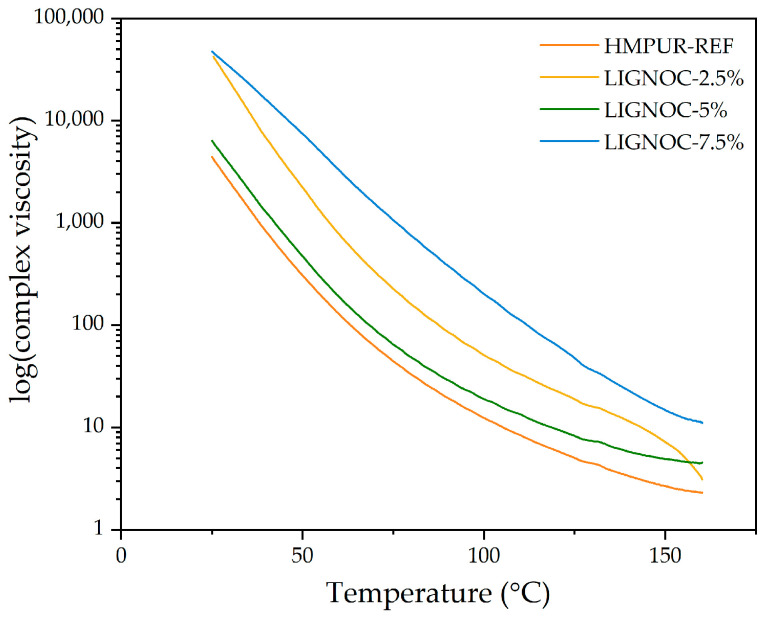
Complex viscosity of the HMPUR adhesives obtained through plate–plate rheometric analysis.

The complex viscosity of the adhesives was also evaluated through plate–plate rheology. As shown in Figure 10, in all cases, the complex viscosities of the new adhesives were higher than that of the reference adhesive.

From Figure 10, it is possible to notice how, in all cases, the complex viscosity decreased logarithmically with temperature, which is typical for polymers as their mobility increases [46,47]. The modification of the reference adhesive with different concentrations of lignin-based polyol increased the complex viscosity in all cases for the temperature range studied. This increase can be attributed to a higher rate of chain interactions due to the lignin-based polyol restricting the mobility of the polymer. It should be pointed out that the LIGNOC-7.5% adhesive had a markedly higher viscosity than the other adhesives throughout the entire range, which could result in difficulties in application using a heated manual gun at lower temperatures, where the difference in viscosities is higher.

**Figure 11:**
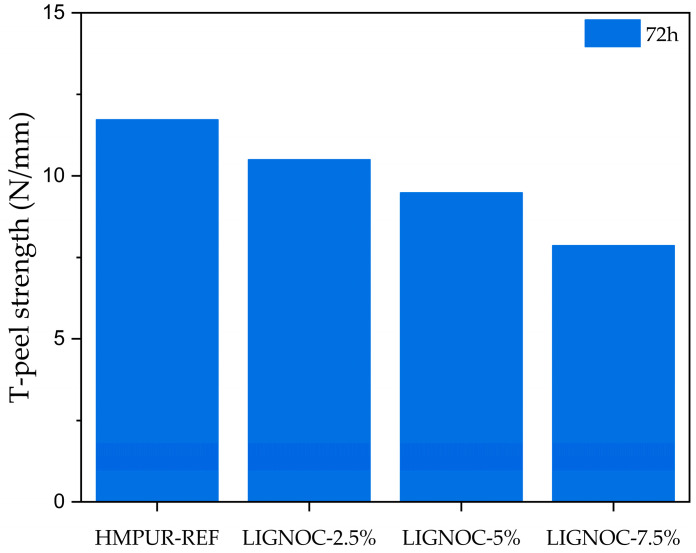
T-peel test results after 72 h from the bonding time.

Finally, T-peel tests were performed to evaluate the adhesion in upper to sole split/adhesive HMPUR/SBR rubber joints—the most demanding test in the footwear sector. Figure 11 shows the results obtained after the peel test.

**Figure 12:**
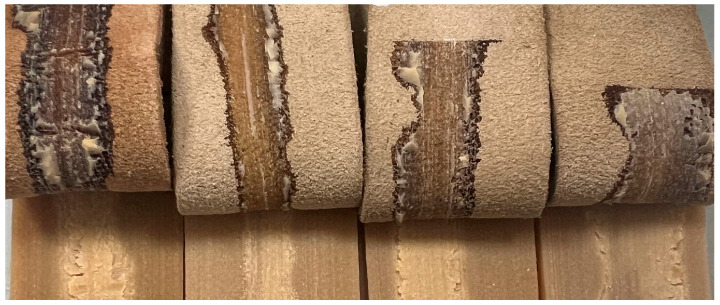
Appearance of samples after T-peel test. From left to right: HMPUR-REF, LIGNOC-2.5%, LIGNOC-5%, and LIGNOC-7.5%.

All adhesives showed a lower adhesive strength than the reference adhesive. However, all of them met the minimum requirements for footwear, according to the standard EN 15307:2015 [48], which indicates a peel strength ≥3.5 N/mm for moderate-/high-demand footwear. Additionally, it is crucial to evaluate not only the peel resistance value but also the nature of the bond; specifically, the appearance of the surface when peeled off, as shown in Figure 12.

Typically, failures in adhesion can be categorised as either adhesion failures, where the separation occurs between two different materials, cohesive failures, where the separation happens within the same material, or a mixture of both types [14]. In this case, both bonding performed with the reference adhesive and the bio-adhesives showed adhesion failure, as determined through the visual checking of the samples after the test, where the adhesive remained on one of the substrates after the separation, concretely separating from the SBR rubber.

## 4. Conclusions

This study provided compelling evidence supporting the advancement of sustainable adhesive production through the use of lignin-based polyols derived from agricultural by-products, such as rice straw. The exploration and utilisation of lignin—an abundant and under-utilised natural resource—in the synthesis of HMPUR has significant environmental and industrial implications.

Our research established that lignin-based polyols can effectively replace a substantial portion of fossil-based polyols without compromising the essential adhesive properties. After successful conversion from rice straw-derived lignin to polyol, the experimental outcomes revealed that the adhesives synthesised with up to 7.5% lignin-derived polyol not only maintained comparable thermal and mechanical properties but, in some instances, showed an enhanced thermal stability and higher residue content after decomposition. This indicates an improvement in the structural integrity and resistance of the adhesives at elevated temperatures. Moreover, the integration of lignin-based polyols accelerated the curing process and increased the glass transition temperature, suggesting the potential for higher performance in applications demanding robust thermal characteristics.

The successful incorporation of lignin-based polyols in HMPUR adhesives represents a significant stride towards reducing the carbon footprint of polyurethane adhesives. Through leveraging bio-based resources, the adhesive industry can diminish its reliance on non-renewable petrochemicals, thus aligning with global sustainability goals. Furthermore, the use of agricultural waste not only helps in waste reduction but also adds value to otherwise low value by-products. The environmental benefits, coupled with the retention and even enhancement of adhesive properties, underscore the potential of lignin-based polyols as a pivotal component in the future of green chemistry.

Looking forward, it is imperative to expand the scope of research to optimise the extraction and modification processes of lignin, in order to enhance its compatibility and performance in adhesive formulations. Additionally, exploring other types of agricultural and industrial wastes as potential sources of lignin could further broaden the resource base. Potential applications could extend beyond the footwear industry, into areas such as the automotive, construction, and packaging industries, in which the demand for strong, durable, and environmentally friendly adhesives has been increasing. Moreover, advancing our understanding of the interactions between lignin-based polyols and other polymer components could lead to innovations in other types of polymers and composites, potentially opening new avenues for the use of sustainable materials in various high-performance applications.

In summary, the adoption of lignin-based polyols in the synthesis of polyurethane adhesives both supports environmental sustainability and presents an opportunity for the adhesive industry to contribute actively to a more circular economy. Continued research and development in this field are crucial to overcoming existing challenges and unlocking the full potential of bio-based polyols in commercial adhesive applications.

## Figures and Tables

**Table 1:** Nomenclature and formulation of synthesised adhesives with PPG and lignin-based polyol percentages and desired free NCO%.

Formulation	Lignin-Based Polyol wt.%	PPG wt.%	Desired Free NCO (%)
HMPUR-REF	-	100.0	3.75
LIGNOC-2.5%	2.5	97.5	3.70
LIGNOC-5%	5	95.0	3.67
LIGNOC-7.5%	7.5	92.5	3.63

**Table 2:** Decomposition temperatures and weight loss data obtained from TGA for the developed lignin-based polyol.

Sample	T1 (°C)	∆w1 (wt%)	T2 (°C)	∆w2 (wt%)	Residue (%)
Lignin-based polyol LIGNOC	140	25	302	37	28

**Table 3:** Obtained conditions of the synthesised adhesives.

Adhesive	Reaction Time (min)	Free Isocyanate (%NCO)
HMPUR-REF	60	3.87
LIGNOC-2.5%	26	3.85
LIGNOC-5%	16	3.68
LIGNOC-7.5%	9	3.35

**Table 4:** Decomposition temperatures and weight loss data obtained from TGA for the HMPUR adhesives.

Adhesive	T1 (°C)	∆w1 (wt%)	T2 (°C)	∆w2 (wt%)	T3 (°C)	∆w3 (wt%)	Residue (%)
HMPUR-REF	334	62	399	33	-	-	7
LIGNOC-2.5%	333	60	400	34	-	-	7
LIGNOC-5%	334	58	399	34	-	-	9
LIGNOC-7.5%	341	50	375	32	438	6	12

**Table 5:** Glass transition temperatures obtained from DSC thermograms of the HMPUR adhesives.

Adhesive	Tg (°C)
HMPUR-REF	−18.86
LIGNOC-2.5%	−17.02
LIGNOC-5%	−18.06
LIGNOC-7.5%	−14.21

## Data Availability

Data are contained within the article.
